# The role of community-based counsellors in increasing TT surgical uptake and enhancing gender equity

**Published:** 2025-03-07

**Authors:** Fortunate Shija, Robert Geneau

**Affiliations:** 1 Kilimanjaro Centre for Community Ophthalmology, Tanzania.; 2 Division of Ophthalmology, University of Cape Town, South Africa.

**Speaking the local language and understanding local social norms allows counsellors to foster trust and alleviate fear.**

**Figure:**
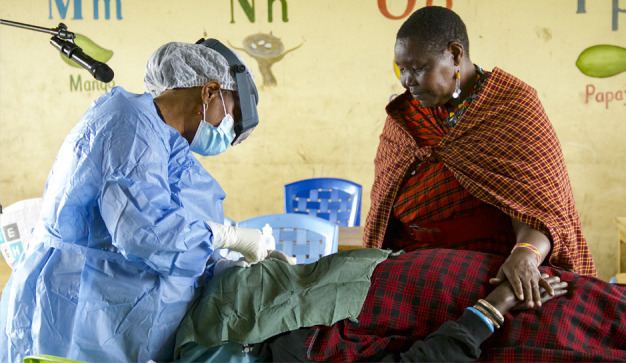
A community-based counsellor (right) offers emotional support to a woman during TT surgery. tanzania

© GERALD THADEUS CC BY-NC-SA 4.0

Trachomatous trichiasis (TT), the blinding stage of trachoma, remains a significant public health problem in remote and underserved areas. Surgery for TT is typically offered free of charge through trachoma outreach campaigns. TT is considered a minor surgical procedure, aimed at correcting in-turned eyelashes to prevent corneal damage and blindness caused by trachoma. Despite this, many programmes have reported some people declining TT surgery, including many women.

In 2022, the Kilimanjaro Centre for Community Ophthalmology introduced a community-based counseling approach in Ngorongoro district, Tanzania, to understand surgery hesitancy, to educate community members about the benefits of TT surgery, and to improve surgical uptake. The district is home to Maasai communities, who have traditionally been less engaged with institutional health care services compared to other groups in Tanzania.

## Understanding surgical hesitancy

Many patients hesitate or decline TT surgery due to deeply ingrained cultural and social concerns. In Ngorongoro, several common reasons for hesitancy were identified, including fear of surgery, concerns about pain or permanent damage, and the burden of household responsibilities, particularly for women. Additionally, many women require spousal approval before undergoing medical procedures, while some patients prefer traditional epilation methods that provide temporary relief, delaying the perceived need for surgery. Travel difficulties, especially among nomadic communities, further exacerbate these challenges and disproportionately affect women.

## Community-based counselling

The integration of a local community-based counselor (CBC) has been instrumental in addressing these concerns and the underlying reasons for surgical hesitancy in Ngorongoro District. Selected from within the community, the CBC is deeply familiar with local customs, languages, and norms. Other selection criteria include strong communication skills, established trust and respect within the community, and prior experience in health or social services. Given that TT disproportionately affects women, a female CBC was selected to facilitate communication with female patients. However, she was trained to provide support to both male and female patients.

A community-based counseling approach starts with a training phase that covers culturally appropriate counseling techniques, active listening, and how to address misconceptions about TT surgery. Once integrated into the trachoma programme, the CBC usually engages with TT patients multiple times and in different locations, such as outreach sites and patients’ homes. The counseling sessions foster a two-way dialogue, allowing patients to voice their fears and questions while the counselor addresses misconceptions, offers reassurance, and supports informed decision-making. The CBC will engage with the decision-makers in the family (usually men) to encourage them to approve surgery for their wives. The CBC also provides pre- and post-surgical emotional support.

## Impact on surgical uptake in Ngorongoro

The introduction of a CBC in 2022 has led to an increase in TT surgery acceptance. Previously, 35 patients in one of the three divisions in the district had declined surgery three times. Between September 2023 and September 2024, 20 of these 35 patients eventually accepted surgery as a result of counselling by the CBC. Patients were also more likely to refer family members and peers after undergoing surgery themselves.

Of the 20 patients, 18 were women. One female patient shared her experience: “I was afraid to have my eyes cut, but when Paulina, the counselor, explained the process in my own language and stood by me during surgery, I felt safe. Now, I tell other women not to be afraid.”

The success of this approach reflects the unique barriers female patients face in accessing care, and the role of CBCs in facilitating discussions around these challenges.

## Beyond trachoma: sustainable eye health integration

While the focus of this study was on TT, the impact of the community-based counseling approach extends beyond trachoma programmes, with CBCs now also being embedded in cataract surgical outreach activities. By embedding counseling roles into existing community health structures and ongoing eye health programmes, we ensure long-term benefits for eye health, even after TT programmes have transitioned into routine eye health services.

The success of community-based counseling in Ngorongoro underscores the importance of investing in people-centered, culturally competent care. Documenting these initiatives is valuable in supporting the transferability of this model – integrating locally trusted CBCs into eye health programmes – to other underserved communities, ensuring that even the most remote populations have access to the eye care they need, and upholding the principle that no one should be left behind.

